# Vertebral rotation measurement: a summary and comparison of common radiographic and CT methods

**DOI:** 10.1186/1748-7161-3-16

**Published:** 2008-11-02

**Authors:** Gabrielle C Lam, Doug L Hill, Lawrence H Le, Jim V Raso, Edmond H Lou

**Affiliations:** 1 Department of Surgery, University of Alberta, Edmonton, T6G 2B7, Canada; 2 Department of Rehabilitation Technology, Glenrose Rehabilitation Hospital, Alberta Health Services, Edmonton, T5G 0B7, Canada; 3 Department of Radiology and Diagnostic Imaging, University of Alberta, Edmonton, T6G 2B7, Canada

## Abstract

Current research has provided a more comprehensive understanding of Adolescent Idiopathic Scoliosis (AIS) as a three-dimensional spinal deformity, encompassing both lateral and rotational components. Apart from quantifying curve severity using the Cobb angle, vertebral rotation has become increasingly prominent in the study of scoliosis. It demonstrates significance in both preoperative and postoperative assessment, providing better appreciation of the impact of bracing or surgical interventions. In the past, the need for computer resources, digitizers and custom software limited studies of rotation to research performed after a patient left the scoliosis clinic. With advanced technology, however, rotation measurements are now more feasible. While numerous vertebral rotation measurement methods have been developed and tested, thorough comparisons of these are still relatively unexplored. This review discusses the advantages and disadvantages of six common measurement techniques based on technology most pertinent in clinical settings: radiography (Cobb, Nash-Moe, Perdriolle and Stokes' method) and computer tomography (CT) imaging (Aaro-Dahlborn and Ho's method). Better insight into the clinical suitability of rotation measurement methods currently available is presented, along with a discussion of critical concerns that should be addressed in future studies and development of new methods.

## Background

Adolescent Idiopathic Scoliosis (AIS) is a lateral and rotational deformity of the spine, predominantly affecting individuals of age 10 to 17. The progression of AIS occurs during the rapid growth stage due to factors still unknown. Traditionally, measurement of Cobb angles was the primary means of quantifying the severity of AIS in scoliotic patients. However, this method is limited to assessment of the spine in the saggital and coronal planes. More current investigation of vertebral rotation in the axial plane has provided better understanding of AIS as a three-dimensional condition.

Recent studies suggest that the coupling relation between vertebral rotation and lateral motion may provide insight into an indicative characteristic of scoliotic spines [1-4]. Other literature has examined rotation of the vertebral column in connection to the etiology of AIS [5,6], as discussed in the Spine-Rib Hypothesis [7] and in evaluating the Neurocentral Junction Hypothesis [8]. Above all, measurement of vertebral rotation is of key significance in the prognosis and treatment of scoliotic curves [9-11]. It may act as an indicator of curve progression, thus being clinically applicable for both preoperative and postoperative assessment [9,12]. The association of vertebral rotation with rib hump has led to techniques that may be applied to school screening programs [13,14]. Furthermore, vertebral rotation measurement is becoming prominent in assisting pre-surgical planning. Inaccurate knowledge of vertebral rotation may lead to unnecessary surgical operations and, in the case of pedicle screws, misplacements that incur risks of spinal cord injury [15]. At the same time, axial rotation has been equally valuable in better understanding the effect of brace treatment or surgical interventions, as evidenced in the studies evaluating Cotrel-Dubousset [16-19] and Harrington instrumentation [20,21].

While numerous methods have been developed to measure axial rotation, the techniques explored most extensively are those involving landmark identification. The position of the spinous process (Cobb method) [22], pedicle shadows (Nash and Moe, Perdriolle, Drerup, Stokes method) [9,10,23-25], or a combination of landmarks (Mehta) [26] in relation to the vertebral body are often clinically used to quantify the extent of vertebral rotation. The methods mentioned above are performed on images obtained using radiography – a technology that, though popular, is limited in various aspects. In the pedicle-shadow offset technique initiated by Nash and Moe, measurements taken from radiographic images only represent a projected, not actual, rotation [27]. Furthermore, the hazardous health implications associated with frequent and prolonged exposure to radiation has been of primary concern for scoliotic patients, who are often undergoing critical growth and developmental stages. Consequently, there has been growing emphasis on developing new technology that does not involve patient exposure to ionizing radiation. Such examples include real-time ultrasound [28,29], the AUSCAN system [30,31], magnetic resonance imaging (MRI) [32-34], and other innovations [35]. The real-time ultrasound method [28,29] used the Aloka SDD 500 portable ultrasound unit (Olympus, Medical and Industrial Equipment Ltd.) to identify the laminae and the rib to determine the rotation while the patient was lying prone on a couch with their forehead supported. Kirby et al [28] reported that the worst estimation on both rib and vertebral rotation were ± 3.6°. The AUSCAN System (AUtomatic SColiosis ANalyser) (BTS Bioengineering Technology & Systems Inc., Italy) is an automatic optoelectronic device that consists of two pairs of CCD TV-cameras, a FPSR (Fast Processor for Shape Recognition) image processor and a specially developed software package for data processing. Twenty seven body landmarks placed on the skin: 19 on the posterior side and 8 on the anterior side, which included the spinous processes from C7 to S1 were used to reconstruct the internal alignment. The vertebral rotation would then be estimated. To use MR images to estimate the vertebral rotation, a specific MRI images technique was need [32]. Using the Birchall et al method [32], the segmental axial rotation was comparable to the conventional CT method. However, MR images were not commonly requested during scoliosis clinic. The Ortelius 800 machine uses a magnetic field fingertip sensor to identify the spinal process and then to reconstruct the internal spinal structure was another non-ionization method. The vertebral rotation could be estimated based on the location of the spinous process; however, no clinical study has concluded that this method was accurate. The aim of these new developments is to reduce the number of false positives referred to scoliosis clinics through school screening programs as well as unnecessary exposure to radiation. Another technology that is becoming increasingly popular for assessing axial rotation is computer tomography. Aaro and Dahlborn [36] and Ho et al [37] developed techniques of rotation measurement from CT images. Despite many advantages of this technology, radiographic methods remain most standard, to which current developing techniques are compared for evaluating their accuracy [27,38-41].

This review encapsulates six common methods of measuring vertebral rotation based on technology demonstrating greatest relevance in present clinical settings: radiography and CT imaging. An assessment of each technique, founded upon accuracy, economic, and health considerations, forms the focus of this paper. Our findings aim to provide better insight into the clinical suitability of presently available rotation measurement methods, as well as to underscore critical concerns that should be addressed in future development of new techniques.

### Summary of Radiographic and CT Methods

**Figure 1:**
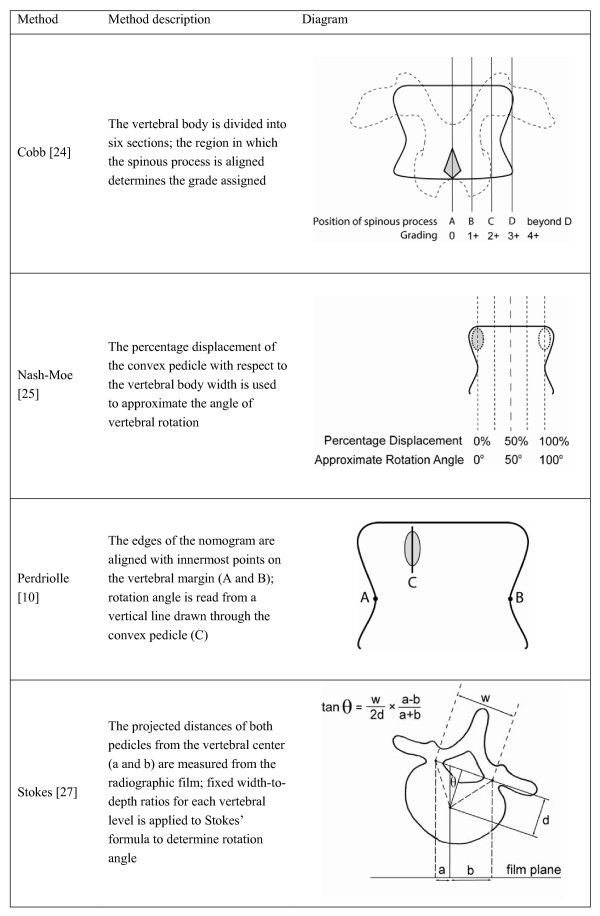
A summary of common radiographic methods of vertebral rotation measurement.

**Figure 2:**
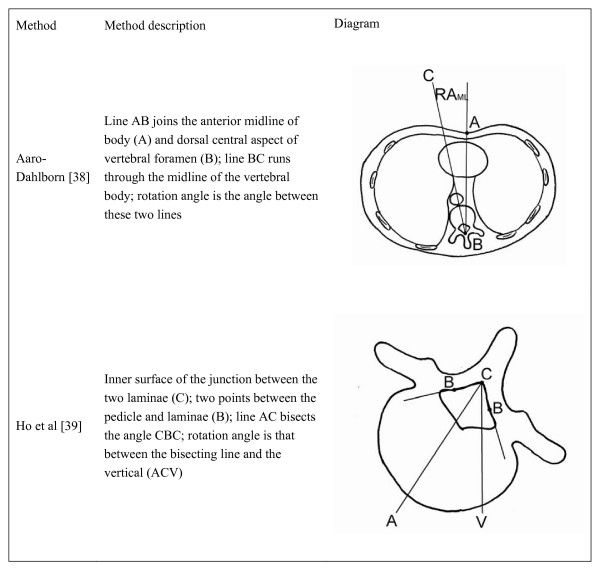
A summary of common CT methods of vertebral rotation measurement.

The following charts (Figures 1 and 2) summarize the radiographic and CT methods of vertebral rotation measurement of interest in this review, respectively. Figure 1 describes and illustrates how the vertebral rotation measured using the Cobb, Nash-Moe, Perdeiolle and Stokes methods with radiographs. Figure 2 describes and illustrates how the vertebral rotation is measured using the Aaro-Dahlborn and Ho et al. methods with CT images. The Cobb method divides the vertebral body into six sections; the region in which the spinous process is aligned determines the grade assigned. The Nash-Moe method describes the percentage displacement of the convex pedicle with respect to the vertebral body width which is used to approximate the angle of vertebral rotation. The Perdeiolle uses the edges of the nomogram to align with innermost points on the vertebral margin (A and B); rotation angle is then read from a vertical line drawn through the convex pedicle (C). When a vertebra rotates, the rotation angle *θ *by Stokes method is $\theta =\tan^{-1}\left(\frac{1}{2}\times \frac{a-b}{a+b}\times \frac{w}{h}\right)$; where a and b are the center of the pedicles relative to the center of the vertebra, respectively; w is the width of the laminar and h is the maximum width of the vertebra from the center to the edge. For the Aaro-Dahlborn method, a line joining the anterior midline of body (A) and dorsal central aspect of vertebral foramen (B) is drawn. Then, a second line runs through the midline of the vertebral body is drawn. The rotation angle is the angle between these two lines. The Ho et al method requires identify the inner surface of the junction between the two laminae (C); two lines are then drawn to join two points between the pedicle and laminae (B). A line (AC) to bisect the angle CBC is drawn. The rotation angle is that between the bisecting line and the vertical line ACV.

### Advantages and Disadvantages of Individual Methods

Radiographic measures are obtained routinely in a standing posture whereas CT measures are obtained in a supine position. It is noteworthy that scoliotic curves appear less severe when supine, both in terms of curvature and rotation. This imperfect relationship complicates comparisons when considering both modalities for longitudinal follow-up of an individual.

#### Cobb method

One weakness of Cobb's method is its ability to provide an approximation of vertebral rotation. The grading scheme is limited to five grades and does not allow quantification of the angle of axial rotation [23]. Nash and Moe later improved on this limitation by suggesting a method of obtaining a rotation degree from the percentage displacement of the landmark with regards to the vertebral body.

Another disadvantage of Cobb's method involves the vertebral landmark used to determine axial rotation. Vertebrae of severe scoliotic cases often exhibit intravertebral rotation, as evidenced by the distortion of the spinous process tip from the frontal center of the vertebral body. The vertebral model referred to in Cobb's study, however, disregarded such asymmetry. Moreover, surgical techniques often alter the spinous process, obscuring visibility following operation. Stokes, Nash and Moe argued that measurements using the spinous process may result in inaccurate apparent rotation [23,25], which motivated their later studies of pedicle shadows to determine vertebral rotation.

Nash and Moe reported the difficulty of visualizing the spinous process on spinal radiographs to be a major challenge [23]. Mehta also discovered the limited visibility of the spinous process problematic in measuring large rotation angles [26]. In addition to the limited visibility of the chosen landmark, Nash and Moe observed inconsistencies in the grading scheme and measurement method. Their study reported a 10–20° underestimation using Cobb's method as well as inconsistencies in the angle interval represented by each grade.

Due to Cobb's method being one of the earliest recorded techniques used for measuring vertebral rotation, most literature focused upon its limitations. Despite these criticisms, the concept of measuring axial rotation using vertebral landmarks from anteroposterior radiographs was adopted by many later methods and remains popular in clinical settings. Cobb's method is valuable in being simple to use and requiring no additional patient exposure to radiation in comparison to more modern methods [42,43]. Moreover, the spinous process is used as a landmark in numerous other improved methods. In 1985, Bunnell developed a measurement method involving the distance of the spinous process with respect to the width of the vertebral body [44]. Drerup also suggested investigating the position of pedicles with relation to the spinous process and vertebral body [9,24]. Both these techniques were found to yield relatively accurate results [45].

#### Nash-Moe method

Nash and Moe claimed their method to be much improved from that of Cobb. One advantage was the better visibility of the particular chosen anatomic landmark over a greater range of angles. Their study was able to investigate rotations of the convex pedicle of up to 90° [23]. Additionally, pedicle shadows could be better seen even after surgery, rendering the method applicable in postoperative assessment. The Nash-Moe method seemed to overcome the concern of intravertebral deformity affecting measurement reliability, which became a major factor in the criticism of Cobb's technique. In comparison to the spinous process, the pedicles are located closer to the vertebral body and, consequently, are not subject to as much distortion in severe scoliotic cases [23,25].

Despite these improvements, there exist some drawbacks of the technique. Foremost, the suggested method of angle determination only provided a rough approximation of axial rotation. Ho observed that grade 0, a neutral position, represented rotation of up to 11° determined by CT scans [37]. Like Cobb's method, the Nash-Moe technique neglected vertebral asymmetries such as non-parallel endplates, concave vertebral walls, and elliptical diameters [9,24]. Other factors overlooked were mentioned by Stokes, including the effect of distance from the x-ray, vertebral body shape and symmetry [25].

The better visibility of pedicle shadows was emphasized as the main advantage of the Nash-Moe method. However, Mehta contradicted this assertion, finding that using a single anatomical landmark limited accurate measurements to small rotations [26]. This same realization was made by the authors of this report, who examined 115 spinal radiographs in two separate sittings. The convex pedicle became difficult to visualize at rotation angles greater than 30°. Furthermore, surgical implantations such as Harrington and Cotrel-Dubousset instrumentation obstructed visibility of pedicles on spinal radiographs. [46,47]

#### Perdriolle method

Conflicting views were presented concerning the accuracy and reliability of rotation measurements performed using the Perdriolle torsion meter. Richards' study [47] found the average individual observer error to be 6°. Only about 50% of observers obtained measurements within 5° of the actual value. Richards also discussed the difficulty of visualizing pedicle landmarks in 20% of postoperative spinal radiographs, for which determination of vertebral rotation is currently most relevant. Another complication involved marking the pedicles on spinal radiographs, where a 2 mm error amounts to 5° of rotation. Richards also recognized that a rotated patient body during radiographic examination further increased measurement error.

Despite Richards' negative findings, most studies highlighted advantages of using the torsion meter. In examining interobserver and intraobserver errors, Barsanti showed that over 92% of errors made were within ± 5° [46]. Omeroglu reached similar conclusions, with 98% of intraobserver measurements within ± 5° [48]. In Weiss' investigation, intraobserver error was reported to be ± 1° and interobserver error ± 3° [49]. These studies also agree that such accuracy can only be observed for mild to moderate rotation, due to difficulty of point selection for vertebrae with large rotation. However, this does not seem to be a limitation, since typical scoliosis incidences involve 15–20° rotation, rarely exceeding 40° [46,49].

Another issue connected to the controversy concerning accuracy and reliability of the torsion meter is its ability to account for irregular vertebral geometry, saggital and coronal inclination. This challenge is more inconclusive, although Weiss noted that their effect on measurement accuracy was insignificant when studying the apical vertebra. He also suggested measuring a second vertebra as reference, so to reduce measurement error due to rotated body position [27]. Overall, many studies reported that the Perdriolle torsion meter is suitable for use in the clinic. It is affordable, non-invasive and simple to use, making it applicable in clinical settings [46,48,50]. Barsanti also highlighted its ability to measure rotation from a single anteroposterior radiograph to be an advantage. In contrast to newly developed techniques like stereoradiography, the torsion meter minimized patient exposure to harmful radiation.

#### Stokes method

Stokes' method accounted for vertebral asymmetry and dimension by determining pedicle-offset with respect to a center point rather than the vertebral edges. His technique simulates biplanar radiography, which involves taking two radiographic images, frontal and lateral, to obtain measurement of vertebral dimensions. Stokes reported similar accuracy, with the added advantage of one fewer x-ray exposure and a less complicated measurement scheme [25].

In a comparative study of four methods used to determine axial rotation from radiographic images [45], the techniques proposed by Bunnell, Drerup, Koreska and Stokes were examined. Results showed close correlation between values obtained by the former three methods. However, Stokes' method demonstrated significant deviation for rotation greater than 5°. Even after Stokes corrected the width-to-depth ratios by a factor of two [51], the results of the comparative study suggested that his method was least accurate among the four techniques tested. Stokes rationalized that using the vertebral center to measure pedicle displacement should be more accurate. However, the higher precision of marking vertebral edges might offset this accuracy. His method incurred least systematic error, but greatest random error.

Another concern regards the use of an averaged width-to-depth ratio for different vertebra levels. The geometry of scoliotic vertebrae can vary greatly among individual conditions. Consequently, these values might be poorly represented in severely distorted vertebrae, affecting accuracy of calculations obtained from Stokes' formula.

#### Aaro-Dahlborn method

A significant advancement of the Aaro-Dahlborn method was its use of CT technology. In comparison with radiography, CT produces clearer and more detailed images. Errors due to poor clarity of vertebral structures, an obstacle for many radiographic techniques, are reduced. Furthermore, in the transverse plane, landmarks are easily seen even with large rotation. This technology overcomes a major challenge faced by radiography: measuring large degrees of rotation.

One major drawback, however, of using CT technology is increased measurement inaccuracy associated with vertebrae inclined in the saggital and coronal planes [11,36,52]. A study by Skalli et al [11] found that in inclined vertebrae with small degrees of axial rotation (less than 10°), the difference between the actual three-dimensional rotation and the projected rotation is relatively small (approximately 2°). Not only does vertebral orientation change with different body positions, scoliotic vertebrae often exhibit rotations in various planes. Therefore, Skalli et al stated that rotation measurements from transverse CT images can be misleading.

Additionally, CT scans require more time and are more expensive than spinal radiographs, limiting its use in clinical settings. If the entire vertebral column is examined, as in radiography, CT involves greater patient exposure to ionizing radiation, particularly in the thoracolumbar region [53]. As discussed earlier, increased exposure to radiation can be particularly harmful for patients undergoing crucial developmental stages.

Consequently, a CT scan of the apex vertebra is conventionally used to indicate rotation severity. CT, however, cannot entirely replace the role of radiography, since a spinal radiograph is still necessary for identification of the apex vertebra. An associated problem was highlighted by one recent study [41], which showed that the apical vertebra did not always exhibit maximum rotation, a challenge in tracing the rotation severity using CT. Furthermore, the supine position required for CT scans also reduces the Cobb angle, rotation angle, and rib hump [36,50]. Measurements taken from CT scans will therefore under represent the true rotation.

The disadvantages discussed above mainly concern the choice of measurement technology. Amongst other CT methods, such as that of Ho et al, the Aaro-Dahlborn method reached measurements with greater correlation to the actual rotation value, even with tilt in the saggital and coronal planes [54]. In a separate study, both Aaro-Dahlborn and Ho's methods showed similar accuracy. The former method was found to be more difficult to use for inexperienced observers [52]. Gocen et al propose that this is due to the less obvious reference points used, such as the anterior midline, in determining RA_ML_. Ho et al reported higher intraobserver and interobserver errors in the use of the Aaro-Dahlborn method when compared to their technique [37].

#### Ho et al method

The method by Ho et al determines rotation from CT scans using the laminae and laminae junction. Their results reported a 95% clinical success rate and 1.2° error ratio [52] compared three methods (Ho et al, Aaro-Dahlborn and Krismer), reaching similar conclusions to that of Ho et al. The former two methods showed closest correlation with the actual rotation value, but Ho et al's method was preferred. The more clearly defined reference points used in this method allowed less experienced observers to reach accurate measurements from the CT images. Their results revealed that Ho's method reduced errors and measurement variability due to reference point selection. While both Aaro-Dahlborn and Ho et al's methods proved clinically applicable and accurate, the study also showed that interobserver reliability was significantly better in the latter technique.

One concern related to axial rotation measurements from CT is the effect of saggital and coronal tilt on accuracy of measurement values, as described earlier. Krismer et al conducted a study [54] investigating the accuracy of Aaro-Dahlborn and Ho et al's methods in measuring axial rotation of vertebra with significant anatomic deformation and rotated in different planes. The measurements obtained using Ho's method showed less correlation to actual values in comparison to the Aaro-Dahlborn method. Krismer et al suggested that the high accuracy found from Ho's study only signifies its applicability in idealistic conditions, not so in clinical settings.

## Discussion

The key advantages and disadvantages associated with each method of vertebral rotation measurement are summarized in the chart below (Table 1). A review of the various methods of measuring vertebral rotation indicates some of the significant hurdles encountered in this area of research. The objective of obtaining accurate measurements seems to be hampered by vertebral irregularities typical of scoliotic cases. Such factors include intravertebral rotation, inclination in different planes, and large rotation angles – the prominence and effect of each being dependent on the particular method used. Although these challenges have long been recognized, a conclusive solution has yet to be reached and remains a primary goal in continuing studies.

**Table 1:** Advantages and disadvantages associated with radiographic and CT methods of rotation measurement

Method	Advantages	Disadvantages
Cobb	▪ Simple procedure	▪ No means to quantify rotation from gradation scheme
	▪ Little patient exposure to radiation (one anteroposterior radiograph)	▪ Distortion of spinous process tip in scoliotic vertebrae may decrease accuracy
		▪ Limited visibility of spinous process on radiographs of vertebrae with large rotation

Nash-Moe	▪ Position of pedicles are less affected by intravertebral rotation; more reliable landmark for measuring rotation	▪ Provides over-estimation of rotation (can be adjusted by 10° as suggested by Drerup)
		▪ Pedicles are poorly visible on vertebrae rotated severely or on spines with surgical instrumentation

Perdriolle	▪ Affordable	▪ Difficulty in making precise markings on radiographs – a 2 mm error corresponds to 5° rotation
	▪ Non-invasive	
	▪ Simple procedure	
	▪ Little patient exposure to radiation (one anteroposterior radiograph)	▪ Reduced accuracy when measuring large degrees of rotation
	▪ General findings report accurate measurements to within ± 5°	

Stokes	▪ Accounts for three-dimensionality of vertebra; similar accuracy to stereoradiograph	▪ Greater random error in comparison to methods involving marking of vertebral edges
	▪ Little exposure to radiation	
	▪ Simple measuring procedure	

Aaro-Dahlborn	▪ Better measurement accuracy even when measuring vertebrae tilted in the coronal and saggital planes	▪ More difficult to use for inexperienced observers due to less obvious landmark definitions

Ho	▪ Clearly defined reference points; simple procedure	▪ Less correlation to actual rotation value when measuring distorted and
	▪ Better interobserver reliability in comparison to Aaro-Dahlborn method when assessing normal vertebrae	tilted vertebrae; less applicable in realistic conditions

Aside from measurement accuracy and precision, clinical applicability of each method is examined in this paper on the basis of its ease of usage, health and financial implications. Radiation exposure is of critical importance in this evaluation, keeping in mind the young age of AIS patients being considered and their vulnerability during crucial growth stages. CT images are insufficient for identifying the apex vertebra, therefore unable to entirely replace the role of radiography in rotation assessment. While radiography yields images with poorer clarity and involves exposure of a larger bodily region to radiation, it entails less radiation exposure overall. Furthermore, accuracy of CT measurements is more severely affected by inclination and tilt, outweighing limitations encountered with radiographic measurement of large rotations when considering realistic conditions. For these two reasons, radiography is still the more clinically suitable alternative.

The literature examined did not allow for entirely transparent conclusions to be made concerning the most clinically applicable rotation measurement method. Many reviews of Perdriolle's torsion meter express consensus in its measurement precision and simplicity of use. Stokes' calculation may also be a promising method. It aims to overcome the difficulty associated with asymmetrical vertebral geometry, accounting for vertebral dimensions in addition to projected distances. However, there presently exist limited studies evaluating Stokes' method, rendering it difficult to reach fair judgments. For instance, the comparative study by Russel et al [45] draws conclusions from testing a small number of vertebrae. Comparison of Stokes' calculations with stereoradiographic techniques may be valuable, since his concept had been motivated by this improved technology. More extensive comparative studies are perhaps an area requiring greater attention in the future, building better groundwork for evaluating methods with potential clinical utility, like that of Stokes.

## Conclusion

Up to this point, there have been many methods proposed to measure vertebral rotation such as Radiography, CT method, ultrasound, MRI and magnetic sensors. Among these, radiography is still the more commonly used method as all orthopaedic surgeons are very familiar with radiographic images. At our site CT and MR images are generally reserved for cases with unusual presentations, neurological symptoms or deficits, rapidly progressing curves and surgical planning for complex congenital cases. The choice between CT and MR rests on the purpose of the intervention with CT scans showing bony detail better whereas MR images are superior for soft tissue identification. As the technology becomes more advanced, and radiation exposure is reduced, either low dose x-ray machines or a better 3D ultrasound imaging machine will be more commonly used to measure vertebral rotation accurately.

## Competing interests

The authors declare that they have no competing interests.

## Authors' contributions

GL conducted the study and prepared the manuscript. DH involved in editing the manuscript. LL conceived of the study. JR conceived of the study. EL conceived of the study, coordinated the project and edited the manuscript. All authors approved the final manuscript.
